# Canine spinal peripheral nerve sheath tumours in 18 dogs (2014–2023): surgical management and long-term outcomes

**DOI:** 10.3389/fvets.2025.1653812

**Published:** 2025-10-01

**Authors:** Jonathan Deacon, Jos Bongers, Catherine Stalin

**Affiliations:** ^1^Moorview Referrals, Newcastle upon Tyne, United Kingdom; ^2^School of Biodiversity, One Health and Veterinary Medicine, University of Glasgow, Glasgow, United Kingdom

**Keywords:** PNST, laminectomy, durectomy, rhizotomy, amputation

## Abstract

Spinal peripheral nerve sheath tumours (PNST) commonly manifest as chronic pain, lameness, and paresis in dogs, and these conditions typically prove resistant to medical management. These tumours present significant surgical challenges due to their anatomical location and the complexity of achieving complete margins whilst minimising post-operative morbidity and complications. This single-centre retrospective study evaluated surgical outcomes in 18 dogs with histologically confirmed PNST treated between 2014 and 2023, examining the effectiveness of both amputation and compartmental resection approaches. An analysis revealed an overall median survival time (MST) of 326 days (range: 28–1,374 days), with three patients remaining alive at study conclusion. Notably, patients achieving R0 proximal margins demonstrated significantly better outcomes with longer overall survival times (range: 311–1,374 days, mean 841 days, median 850 days) compared to those with R1 margins (range: 28–1,357 days, mean 346 days, median 217 days). These findings demonstrate that surgical interventions, particularly when achieving non-infiltrated proximal margins, can provide meaningful improvements in both patient comfort and survival time for dogs affected by PNST.

## Introduction

Peripheral nerve sheath tumours (PNST) are a subset of soft tissue sarcomas arising from either Schwann cells, modified Schwann cells, intraneural fibroblasts, or perineural cells (1–3). They are relatively uncommon and account for only 2% of all canine neoplasia and only 27% of neural tissue tumours (4). In 2021, the World Health Organisation (WHO) listed the cranial and paraspinal nerve tumour group within the CNS tumour classification scheme with benign (BPNST) and malignant (MPNST) subclassifications (5). Histopathological differentiation of BPNST grade 1 (historically schwannoma, neurofibroma, and perineurioma) and MPNST grades 2 and 3 is based on morphology, mitotic index, necrosis, and immunohistochemistry although there are no distinct molecular markers (1–3, 6, 7). An updated histological grading system for human PNST from the Federation Nationale des Centres de Lutte Contre Le Cancer (FNCLCC) has been used in recent studies (6, 8), and a modified version of the system has been proposed for the evaluation of canine PNST for future prospective studies (7, 8).

There are limited treatment options for PNST, and the only reported effective treatment is local control with complete surgical resection to achieve negative margins (6, 9–11). Surgical approaches used were via a craniolateral approach to the brachial plexus (12), a modified lateral approach to the cervical spine (13) a paramedian approach to the thoracolumbar spine (12), a forequarter amputation (14), a pelvic limb amputation by coxofemoral disarticulation (14), a cranial external hemipelvectomy (15), or a caudal external hemipelvectomy (15).

Whilst there are several individual case reports (16–21) and short case series (11, 22–24) of surgical management in the veterinary literature reporting surgical outcomes, histopathological diagnosis, and survival data, there are only four studies with more than 10 cases each. They comprise heterogeneous inclusion criteria and objectives with 51 cases from 1995, prior to the routine use of CT and MRI (9), 16 cases from 2017 evaluating compartmental resection with limb preservation (10), 18 cases from 2017 evaluating treatment involving surgical monotherapy, adjunctive radiotherapy, or radiation monotherapy (25), and 30 cases from 2023 evaluating surgical and oncologic complications (26). All were multicentre retrospective studies. The median survival times ranged from 14 to 4,639 days, reflecting the significant variability in lesion location, diagnostic imaging, and surgical management.

This single-centre retrospective study utilises, where possible and appropriate, the same classification and grading systems as those in previous studies. It may then be possible in future prospective studies to generate a larger, homogenous dataset to allow for more robust formal statistical analysis. The primary aim of this retrospective study is to evaluate clinical outcomes and survival times in dogs undergoing surgical management for PNSTs. Specifically, the study aims to characterise both perioperative and long-term outcomes, including complications and recurrence rates. The secondary objective includes identifying prognostic factors associated with survival time and clinical outcome. The hypothesis is that clean (R0) surgical margins and lower-grade lesions correlate positively with survival times, as is the case in multiple human studies (6).

## Materials and methods

### Case selection

This was a single-centre retrospective cohort study. Medical records at Moorview Referrals from 2014 to 2023 were evaluated. The inclusion criteria were dogs with a histologically confirmed PNST managed by compartmental resection or amputation, with or without a concurrent laminectomy. Cases were excluded if clinical information, imaging findings, surgical description, or a definitive patient outcome with a minimum follow-up duration of 12 month was not available. Client consent was obtained for data inclusion. Due to the retrospective nature of the study, formal Animal Ethics Committee approval was not undertaken.

### Medical records review

Data for each dog were recorded, including breed, sex, age, presenting clinical signs, duration of clinical signs prior to imaging diagnosis, imaging findings, anatomical location, surgical treatment, adjuvant management, length of hospitalisation, complications, histopathological grade, histopathological margins, post-operative limb function, time to recurrence of clinical signs (disease free interval, DFI), and overall survival time (OST).

### Diagnostics

Computed tomography (CT) studies were obtained on a 16-slice Toshiba Aquilion or 80-slice Cannon Lightening SP with the patients in sternal recumbency with the thoracic limbs extended. A minimum of 1.0/0.8 volumetric series in bone and soft tissue algorithms with a minimum of a venous phase computed tomography angiography (CTA) were acquired. Magnetic resonance imaging (MRI) studies were obtained with either a 1.5 Tesla Phillips or 1.5 Tesla Cannon Vantage system. All studies contained at minimum three-plane T2W-sequences and two-plane T1W sequences, with pre- and post-gadolinium contrast. All imaging studies were evaluated by an European College of Veterinary Neurology (ECVN) resident in training (JD), as primary clinician or primary surgeon.

### Surgery

Where individual or two nerve roots of a plexus were affected by imaging, a compartmental resection was performed. If the imaging lesion or grossly abnormal nerve on surgical exploration extended to the neuroforamen, a laminectomy, a durectomy, and a rhizotomy were performed as previously described. In cases where nerves involving multiple affected roots were identified, a limb amputation was performed as previously described (12, 13).

### Complications

Complications were retrospectively graded as intra-operative and post-operative.

Intra-operative complications were defined using the classification of inter-operative complications criteria (CLASSIC) (see Table 1) (27).

**Table 1:** CLASSIC classification for intra-operative complications.

Grade 0	No deviation from the ideal surgical course
Grade I	Deviation from the ideal course with no additional treatment
Grade II	Deviation from the ideal course requires additional treatment but is not life-threatening or leading to permanent disability
Grade III	Deviation from the ideal course with a threat to life and/or permanent disability
Grade IV	Patients died during surgery

Post-operative complications were defined using the Clavien-Dindo classification scheme (28) and were considered post-operative if they occurred after recovery from general anaesthesia. Complications were determined as any deviation from the standard post-operative management (see Table 2).

**Table 2:** Clavien-Dindo classification for post-operative complications.

Grade I	Any deviation from the normal post-operative course *without* the need for pharmacological treatment or surgical, endoscopic, and radiological interventions. The allowed therapeutics include antiemetics, antipyretics, analgesics, diuretics, electrolytes, and physiotherapy
Grade II	Requiring pharmacological treatment with therapeutics other than those in grade 1, including blood transfusions and parenteral nutrition
Grade IIIa Grade IIIb	Requiring surgical, endoscopic, or radiological intervention but not under general anaesthesia Requiring surgical, endoscopic, or radiological intervention under general anaesthesia
Grade IVa Grade IVb	Life-threatening complications requiring immediate or intensive care – single-organ dysfunction Life-threatening complications requiring immediate or intensive care – multi-organ dysfunction
Grade V	Death of a patient

### Histopathology

The complete nerve mass lesion and surgical margin were excised in each case and fixed in 10% buffered formal saline prior to submission. The proximal and distal margins were tagged with sutures, and the submission form was annotated to ensure accurate orientation. Histological diagnosis was performed by a board-certified pathologist on haematoxylin and eosin-stained sections according to the grading system current at the time of each respective surgery. For the purpose of comparison with other current and future studies, the results have been reclassified according to a modified French (Federation Nationale des Centres de Lutte Contre Le Cancer, FNCLCC) grading system on the basis of tumour differentiation, mitotic count, and tumour necrosis from the description in the original pathology reports (see Table 3) (7, 8).

**Table 3:** Modified histological grading, FNCLCC classification.

Tumour differentiation	
Score 1 Score 2 Score 3	Closely resembling normal tissue Histological typing is certain Embryonal or undifferentiated sarcomas
Mitotic count (per 2.37 mm^2^)	
Score 1 Score 2 Score 3	0–9 mitoses per 2.37 mm^2^ 10–19 mitoses per 2.37 mm^2^ >19 mitoses per 2.37 mm^2^
Tumour necrosis	
Score 0 Score 1 Score 2	No necrosis <50% necrosis >50% necrosis
Histological grade	
Grade 1 Grade 2 Grade 3	Total score 2,3 Total score 4,5 Total score 6,7,8

Histologic margins were defined based on the traditional residual (R) classification system based on the pathology and surgery reports (see Table 4) (29).

**Table 4:** Residual tumour (R) classification.

R0	No residual tumour
R1	Microscopic residual tumour
R2	Macroscopic residual tumour

### Pre- and post-operative limb function

Data on limb function were acquired from the medical records. In patients exhibiting a lameness or lower motor neuron paresis (C6-T2, L4-S1), pre- and post-operative limb function was assessed using a modification of the Arnoczky and Tarvin grading schedule and retrospectively graded from the clinical description to allow for comparison (see Table 5) (10).

**Table 5:** Arnoczky—Tarvin lameness grade.

Grade 0	No lameness
Grade I	Minimally impaired locomotion
Grade II	Impaired locomotion, still weight bearing
Grade III	Lameness, infrequent non-weight-bearing
Grade IV	Non-weight-bearing lameness

In patients with upper motor neuron lesions (C1-C5, T3-L3), pre- and post-operative neurological grades were recorded using the modified Griffiths’ classification (see Table 6) (12).

**Table 6:** Modified Griffiths neurological grade classification.

Grade 0	Normal
Grade I	Cervical or thoracolumbar pain, hyperaesthesia
Grade II	Paresis (muscle weakness) with decreased proprioception, ambulatory
Grade III	Severe paresis with absent proprioception, not ambulatory
Grade IV	Paralysis, decreased or no bladder control, and conscious deep pain perception present
Grade V	Paralysis, urinary and faecal incontinence, no deep conscious pain perception

### Survival and prognostic factors

Follow-up data for DFI and OST were acquired from the medical history and direct client communication.

## Results

### Case information

**Figure 1:**
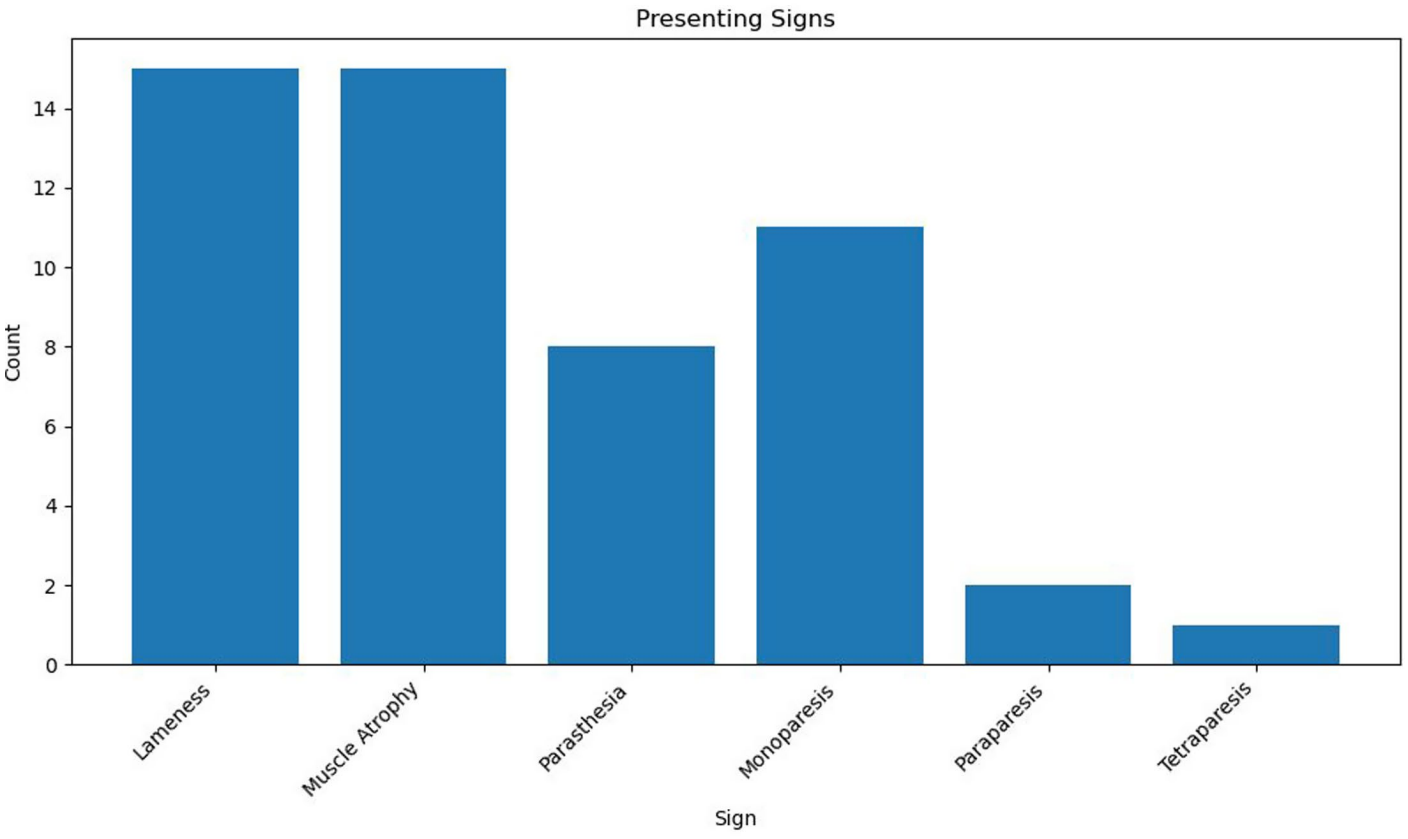
Patient presenting signs.

Eighteen cases met the inclusion criteria. Due to the relatively small sample size, descriptive statistical analysis was considered most appropriate for the majority of variables. Where appropriate Kaplan–Meier plots and log-rank tests were performed using XLSTAT (Microsoft Excel). Breeds represented included Labrador Retriever (*n* = 8, 44%), Bedlington Terrier, Belgian Shepherd Dog, Bichon Frise, Border Terrier, Cocker Spaniel, Crossbreed, Dandie Dinmont Terrier, Rough Collie, Springer Spaniel, and West Highland White Terrier (*n* = 1, 5.6%). Of the 18 dogs, 10 were female (56%), all of which were neutered, and 8 were male (44%), 5 were entire (28%), and 3 were neutered (17%). The median age was 8 years 2 months (range 6 years 5 months–12 years 3 months), and the median body weight was 20.9 kg (range: 5.9 kg–38 kg). Presenting clinical signs were lameness (*n* = 15), muscle atrophy (*n* = 15), paraesthesia (*n* = 8), monoparesis (*n* = 11), paraparesis (*n* = 2), and tetraparesis (*n* = 1). The median duration of clinical signs noted by the client, prior to referral presentation, was 49 days (range 2 days to 210 days) (see Figure 1).

### Imaging results

Diagnostic imaging of the mass was performed by MRI (*n* = 12), CTA (*n* = 6), or CT myelography (*n* = 2). All patients had CTA assessments for distant metastasis, which was negative in all patients (*n* = 18). Local lymph node enlargement was not evident in any of the dogs. Location was intracanal (*n* = 2), extraforaminal/peripheral (*n* = 8), or a combination (*n* = 9).

**Figure 2:**
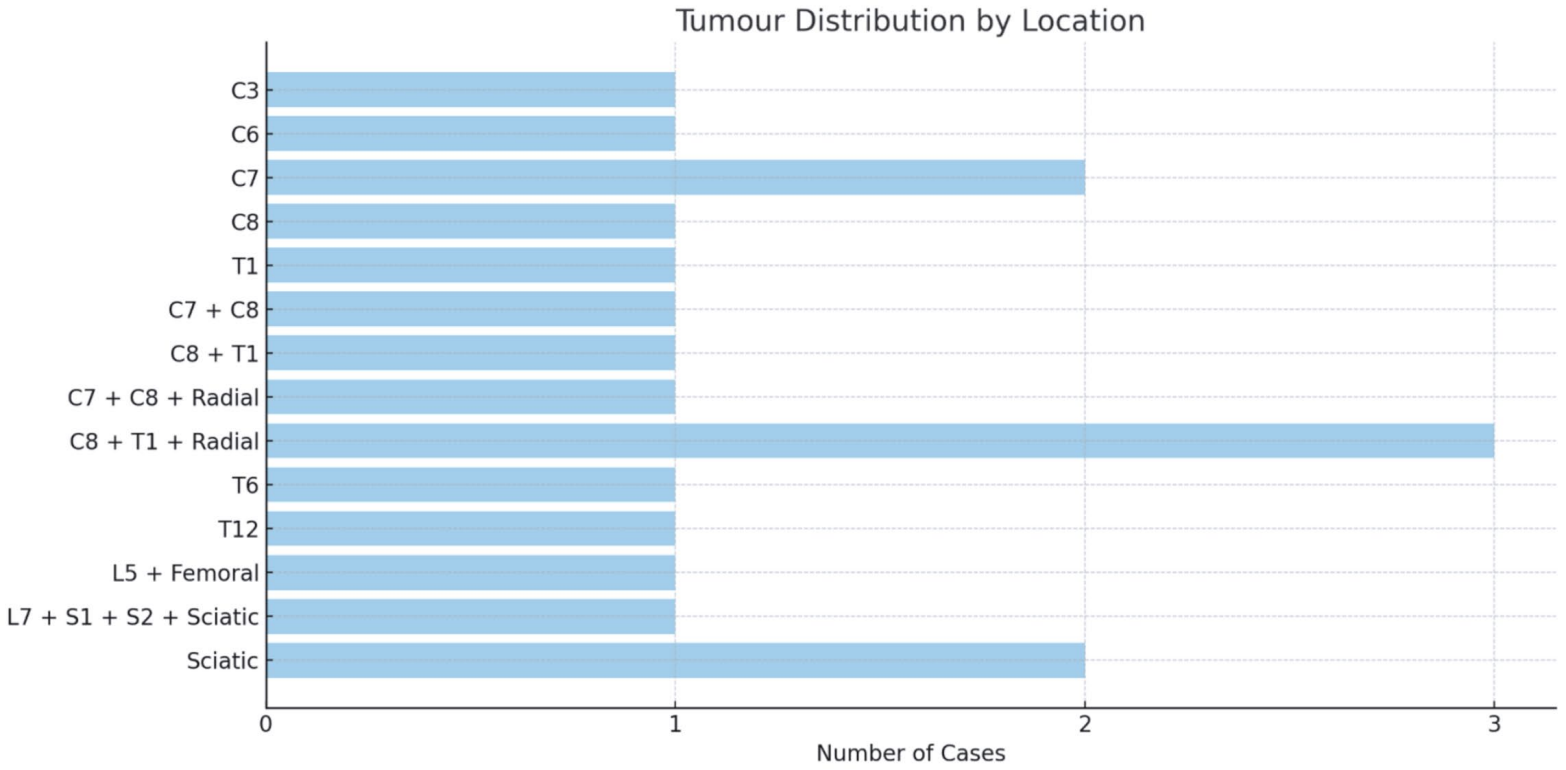
Lesion distribution.

The brachial plexus was involved in 61% (*n* = 11), the lumbosacral plexus in 22% (*n* = 4), and the non-plexus nerves in 17% (*n* = 3) of patients. Lesion size varied considerably and is recorded in Supplementary material (see Figure 2).

### Surgery

Surgeries performed were compartmental resection with laminectomy, durectomy and rhizotomy (*n* = 9), forequarter amputation with laminectomy, durectomy and rhizotomy (*n* = 2), forequarter amputation (*n* = 2), compartmental resection (*n* = 2), hemipelvectomy (*n* = 2), hemipelvectomy with laminectomy (*n* = 1), and pelvic limb amputation with laminectomy (*n* = 1). A total of 19 surgeries were performed on 18 dogs. Patient 2 had a compartmental resection, laminectomy, durectomy, and rhizotomy of C8 initially with a return of clinical signs at 258 days. CT myelography documented an intradural extramedullary compression at C7. Revision surgery was performed as a second compartmental resection, laminectomy, durectomy, and rhizotomy.

Single spinal nerves were implicated in 9 out of 18 patients, two spinal nerves in 6 out of 18 patients, and three spinal nerves in 1 out of 18 patients. The imaging and/or intra-operative mass effect extended from the spinal nerves into a named nerve in 6 out of 18 patients (radial *n* = 4, sciatic *n* = 1, and femoral *n* = 1) or arose solely in the named nerve after the confluence of the plexus roots in 2 out of 18 patients, both of which were in the sciatic nerve.

### Adjuvant management

Three patients (2, 3, and 4) received adjuvant metronomic chemotherapy with meloxicam (Metacam, Boehringer Ingelheim) and cyclophosphamide (Endoxan, Baxter Healthcare).

One patient (11) received adjuvant hyper-fractionated radiation with 20×2.5Gy fractions for a total treatment of 50 Gy.

Two patients (1 and 2) with radial nerve deficits and carpal extension deficits had improved limb use with thermoplastic moulded splint supports. One (patient 1) subsequently had pancarpal arthrodesis with a significant improvement in limb use.

### Hospitalisation

The length of post-operative hospitalisation ranged from 1 day to 8 days, with a mean of 2.4 days and a median of 2 days. Patients with longer durations of hospitalisation (5, 6, and 8 days) were those with higher Modified Griffiths grades (grade 3 and 4) pre-operatively or with transient post-operative neurological deficits following durectomy and rhizotomy (patients 11, 2, and 6, respectively). Patients undergoing amputation (*n* = 8, thoracic limb = 4, pelvic limb = 4) were discharged between 1 and 3 days. There was no difference between thoracic and pelvic limb hospitalisation times.

### Complications

Surgical complications were recorded in five dogs (28%). An intra-operative complication was recorded in one dog (5.6%). Patient 9 suffered a cardiorespiratory arrest non-responsive to resuscitation at the time of surgical site closure following a C5-6 hemilaminectomy, durectomy, and rhizotomy for a C6 nerve mass (CLASSIC grade IV). Four dogs experienced post-operative complications (22%). Patients 7 and 10 experienced transient urinary incontinence that resolved without treatment (Clavien-Dindo grade 1). Patient 5 experienced a suspected UTI based on sediment cytology that resolved with co-amoxiclav (Synulox, Zoetis). Patient 14 experienced a confirmed UTI with isolates of *Escherichia coli* and *Enterococcus faecalis* (Clavien-Dindo grade 2) that resolved with co-amoxiclav (Synulox, Zoetis).

### Histopathology

**Figure 3:**
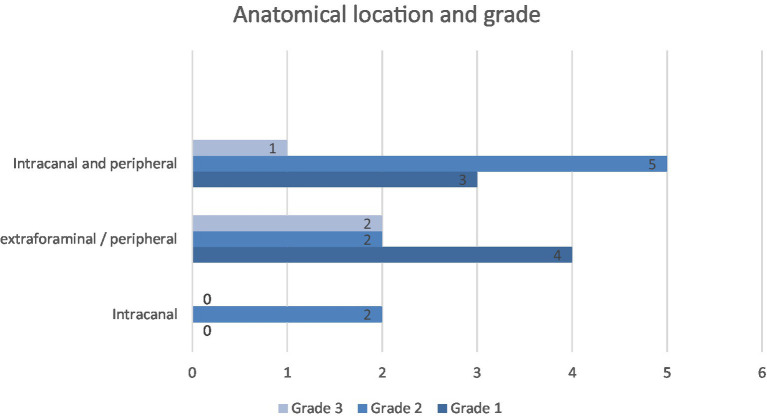
Anatomical location and grade.

A total of 19 sample submissions from 18 patients were classified as PNST (patient 2 had a compartmental resection of C8 with a R0 proximal and R1 distal margin with a second compartmental resection of C7 at 270 days post-operative). Tumours were classified as grade 1 (*n* = 7, 37%), grade 2 (*n* = 9, 47%), and grade 3 (*n* = 3, 16%) (see Figure 3).

**Figure 4:**
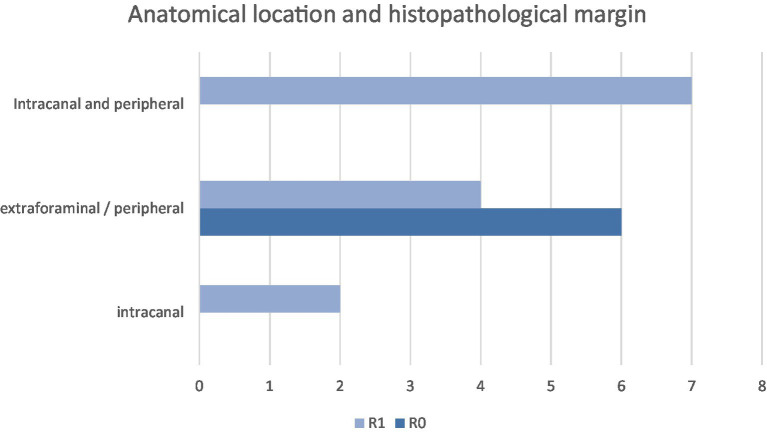
Anatomical location and histopathological margin.

Histologic margins were reported as R0 (*n* = 6, 32%) and R1 (*n* = 13, 68%). In the R0 group (*n* = 6), all patients had amputations (pelvic limb *n* = 3, thoracic limb *n* = 3). One of the thoracic limb amputations had concurrent hemilaminectomy, durectomy, and rhizotomy at C7 and C8 (patient 8) (see Figure 4).

### Pre- and post-operative limb function

Pre-operative limb function was grade 2 (*n* = 4), grade 3 (*n* = 9), and grade 4 (*n* = 2) for the plexus group (modified Arnoczky and Tarvin grading schedule). In the non-plexus group (*n* = 3), neurological severity was grade 2 (*n* = 1), grade 3 (*n* = 1), and grade 4 (*n* = 1) (modified Griffiths’ classification). In the nine patients that did not undergo amputation (*n* = 8) or died (*n* = 1), post-operative limb function was considered stable in 33% (*n* = 3) and improved in 67% (*n* = 6) of patients. In the non-plexus group, all patients (*n* = 3) improved to grade 0.

### Survival and prognostic factors

Of the patients surviving to discharge (*n* = 17, 94%), the median OST was 326 days (range 28–1,374 days, mean 521 days). The 1-, 2-, and 3-year calculated survival rates were 47% (*n* = 8), 29% (*n* = 5), and 18% (*n* = 3), respectively. Nine patients were euthanised due to suspected recurrence (*n* = 4), confirmed local recurrence (*n* = 4), and failure to improve (*n* = 1). Recurrence was confirmed by CTA (*n* = 2) and CT myelo (*n* = 2). Of the five patients that died during the study period for non-tumour-related causes, four were euthanised for other reasons, and one (patient 11) died at home at 157 days post-operative with no clinical signs attributable to local recurrence. Three patients are still alive at the time of submission (1217-, 961-, and 443-days post-operative to date). One patient is lost to long-term follow-up.

**Figure 5:**
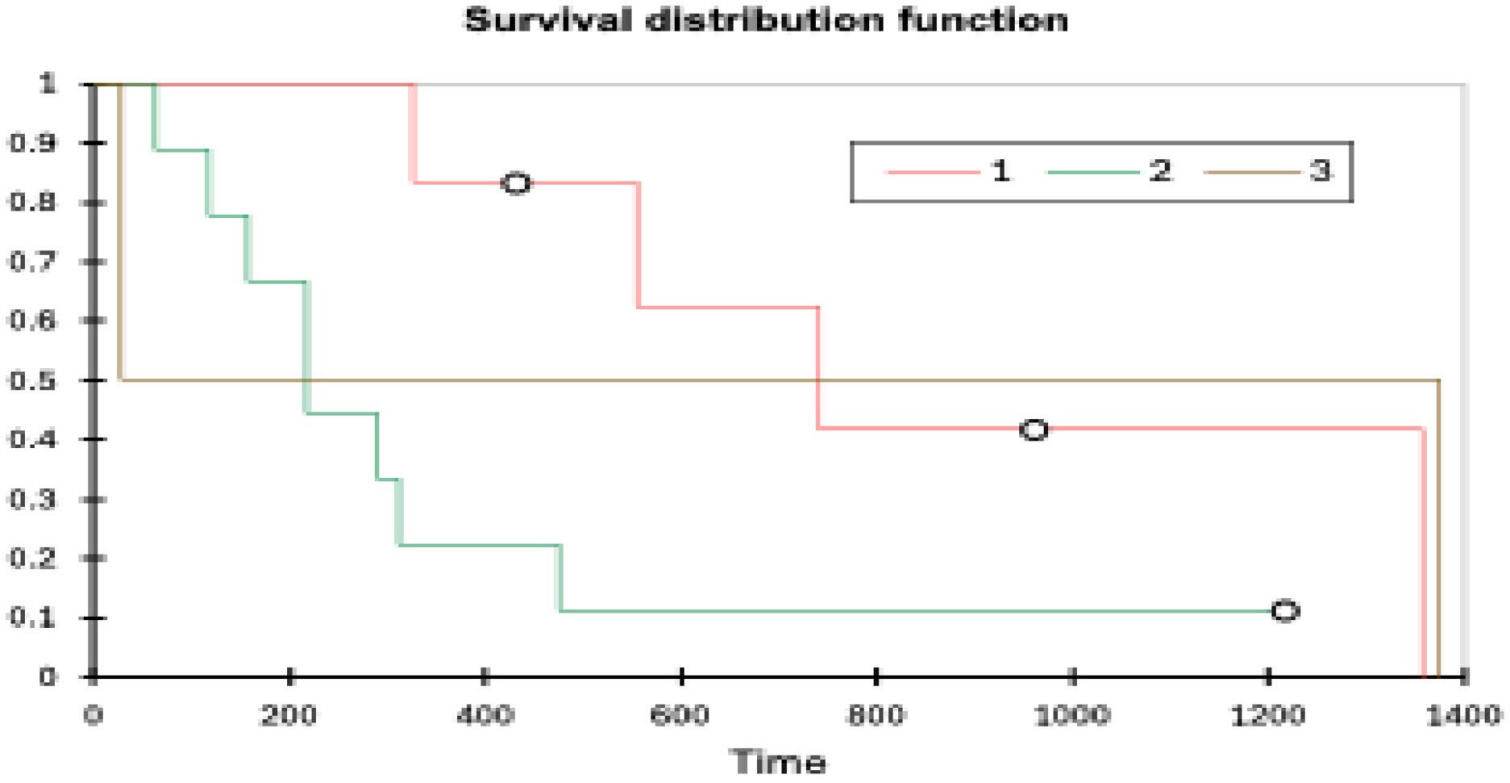
Kaplan–Meier plot of histological grade and OST (XLSTAT, Microsoft Excel).

Patients with grade 1 tumours (*n* = 6) had the longest OST (range 326–1,357 days, mean 890 days, median 648 days) followed by grade 3 tumours (*n* = 2) (range 28–1,374 days, mean 701 days, median 701 days) and grade 2 tumours (*n* = 9) (range 64–1,217 days, mean 258 days, median 217 days). A log rank test (XLSTAT, Microsoft Excel) showed a *p*-value of 0.099 (significance *p* < 0.05). Tumour grade did not affect the OST (see Figure 5).

**Figure 6:**
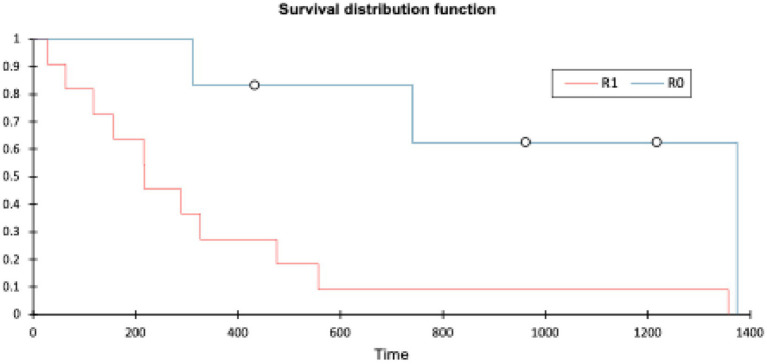
The Kaplan–Meier plot of histological margin and OST (XLSTAT, Microsoft Excel).

Patients with an R0 margin had a longer OST (range: 311–1,374 days, mean 841 days, median 850 days) than patients with an R1 margin (range: 20–1,357 days, mean 346 days, median 217 days). The log-rank test (XLSTAT, Microsoft Excel) showed a *p*-value of 0.007 (significance *p* < 0.05). Histopathological margin did have a significant effect on OST (see Figure 6).

## Discussion

This study describes the cases of 18 dogs from a single institution with histologically confirmed PNST that were treated with surgical management with a definitive outcome or a minimum of 12-month follow-up. Findings were assessed according to the classification systems proposed in previous studies to allow for a direct comparison. Pre-operative lameness and neurological status were graded using the Arnoczky and Tarvin and the modified Griffiths’ classifications, as suggested by L van Stee et al. (10) Complications were graded according to the CLASSIC and Clavien-Dindo classification systems, in line with the study of Stokes et al. (23) and histological findings were updated in line with the modified FNCLCC grading, as suggested by Tekavec et al. (7) and Morabito et al. (8).

This study suggested an overrepresentation of Retriever breeds (44%), a trend also observed in previous research where Retrievers accounted for 40% (30), 38% (10), 33% (31), 27% (26), 22% (32), and 20% (9) of cases. This pattern may reflect a selection bias influenced by the breed’s popularity in the hospital’s geographic area, rather than a true increase in disease incidence, as the proportion of Retriever patients among PNST cases has not been compared to their overall representation amongst all hospital admissions. However, a known predisposition for soft tissue sarcomas in Retriever breeds may also contribute to their higher representation in PNST cases (33, 34).

Peripheral nerve sheath tumours (PNSTs) present with a wide range of clinical signs, depending on the specific nerve affected and its anatomical location. In this study, lameness and muscle atrophy were the most common presenting signs (both observed in 15 cases), particularly in dogs with involvement in the brachial or lumbosacral plexuses. These signs were typically associated with a longer duration of symptoms prior to referral, ranging from 21 to 210 days. In contrast, patients exhibiting upper motor neuron signs such as tetraparesis (C1–C5 lesions) or paraparesis (T3–L3 lesions) tended to present earlier, with symptom durations ranging from 2 to 60 days.

Although there was a slight trend for higher grade tumours to have a shorter survival, it was not statistically significant (*p* = 0.099). There was a large amount of overlap between the grades, and the sample sizes were very small (grade 1 *n* = 7, grade 2 *n* = 9, grade 3 *n* = 3). Notably, one of the three grade 3 patients, a peripheral lesion with an R0 margin, survived for 1,374 days, representing a significant outlier, which had an effect, but this study does highlight that even high-grade lesions can be successfully surgically managed. Grade distributions in this study were similar to those seen in other studies (grade 1–36%, grade 2–43% and grade 3–20%) (8) and histological grade has also previously been reported to not correlate with survival time (10, 26).

Histological margin status had the most significant impact on both disease-free interval and overall survival time, with a difference in median survival of 633 days between groups. A log-rank test comparing R0 and R1 margins gave a *p*-value of 0.007, consistent with a significant difference (*p* < 0.05). This finding is similar to a previous study involving 16 dogs undergoing compartmental resection, where surgical margins were the only statistically significant prognostic factor for survival; dogs with clean (R0) margins had a markedly longer median survival time (MST 2227 days) compared to those with incomplete (R1) margins (MST 487 days) (10). In both that study and the present cohort, histological grade did not influence the likelihood of achieving clean margins. Similarly, a separate study of 30 dogs found no correlation between histological grade and margin status but also did not identify a significant association between margin status and survival time (26).

In this study, the type of surgery, largely dictated by tumour location, was strongly associated with surgical margin status. Amongst the 13 cases that underwent laminectomy, only one achieved clean (R0) margins; the remainder had R1 proximal margins, with one exception having an R1 distal margin. This finding aligns with previous studies reporting spinal canal involvement as a negative prognostic factor due to the anatomical constraints that limit wide excision (8, 10, 31). However, one study did not find a correlation between tumour location, surgical approach, and margin status (26). All dogs in the R0 margin group (*n* = 6) underwent limb amputation (three pelvic, three thoracic), although only two of these also had laminectomies. Notably, two additional dogs that underwent both amputation and laminectomy still had R1 proximal margins. Interestingly, two-compartmental resections did not achieve clean distal margins, suggesting that more aggressive surgery is required to dissect the distal nerve away from the surrounding tissue. This may not be because the surgery is challenging, but may reflect a lack of awareness of what the length of nerve that should be taken distal to grossly abnormal tissue to ensure adequate margins. A follow-up study assessing nerves from amputated limbs could be useful in investigating this.

The decision on which surgical management option was most appropriate was dependent on the lesion location, patient morbidity, and the client’s expectations and preferences. In patients with lesions <3 cm from the spinal cord on imaging or intra-operative assessed gross margins a laminectomy, durectomy, and rhizotomy were recommended. In patients with an extraforaminal/peripheral lesion with an imaging or intra-operative assessed margin of >3 cm distal to the neuroforamen, compartmental resection or amputation was recommended. This appears to be an appropriate decision, as all cases that did not have a laminectomy achieved proximal margins. Amputation was either the clients’ preference or strongly recommended if the distal resection comprised the radial, sciatic, or femoral nerve and would severely impair function. Additionally, this seems appropriate given that all but one dog that did not have amputation showed improvement or stability in the Arnoczky-Tarvin and Modified-Griffiths grades. In addition, patients with continuing mechanical lameness due to carpal hyperflexion benefited from carpal support by an external splint or pancarpal arthrodesis. Importantly, all patients were subjectively more comfortable following surgical resection at least until the point of documented recurrence, highlighting the potential palliative benefit of surgical management even in cases where recurrence is still highly likely.

Some early cases in the series received metronomic chemotherapy with meloxicam (Metacam, Boehringer Ingelheim) and cyclophosphamide (Endoxan, Baxter Healthcare), although this was not subsequently advised routinely due to the low evidence base for it increasing the MST (35) and the lack of observed benefit for the patients in this cohort.

In human PNST management, adjuvant radiation is recommended for local control following wide surgical excision or particularly in the event of compromised or incomplete surgical margins. One patient with an R1 proximal margin from a C3 nerve resection received adjuvant hyper-fractionated radiation at a tertiary centre. The patient made a full neurological recovery from tetraparesis to a normal neurological examination following surgical resection and subsequently received 50Gy in 20 fractions. The patient unfortunately died in his sleep at home, 157 days post-op, with no preceding clinical signs, due to undetermined causes, so the long-term effect on local recurrence could not be assessed. Adjuvant radiation therapy was advised and offered to all patients with incomplete margins but generally declined by the clients on logistical grounds. A recent retrospective study looking at the use of post-operative stereotactic radiotherapy following incomplete surgical margins documented a significantly increased survival time when the SRT treatment was delayed until signs of recurrence were noted rather than in an immediate post-operative setting (25). Further investigation in this area would be beneficial to determine optimal patient management options.

Of the eight limb amputations, including three hemipelvectomies, no intraoperative complications were recorded. One CLASSIC grade IV intraoperative complication was recorded in a patient undergoing a compartmental resection of a C6 nerve, including minihemilaminectomy, durectomy, and rhizotomy, with the patient suffering a cardiorespiratory arrest non-responsive to resuscitation (*n* = 1, 5%). Four dogs (22%) experienced post-operative complications, two grade 1 and two grade 2 on the Clavien-Dindo classification, all of which resolved with treatment. This finding correlates favourably with a previously reported intraoperative complication rate of 13% and post-operative complication rate of 37% for surgical management of brachial and lumbosacral plexus PNST (26).

Of the patients surviving to discharge (*n* = 17, 94%), the median OST was 326 days (range 28–1,374 days, mean 521 days). This finding correlates closely with an OST of 536 days in a study looking at amputation for plexus tumours (26) but is markedly less than the 1,303 days reported in a cohort of 16 dogs with compartmental resection and limb preservation (10). This longer OST likely reflects a larger percentage of clean margins (56% vs. 33%) and fewer proximal lesions requiring laminectomy (38% vs. 72%). There is still a difference in median survival time for dogs with R0 margins (2,227 days vs. 850 days); however, all the cases were dead at analysis in the initial paper, whereas half the patients with R0 margins are still alive in this study, and therefore, the final median survival time will be longer.

The 1-, 2-, and 3-year calculated survival rates were 44% (*n* = 7), 29% (*n* = 5), and 18% (*n* = 3), respectively. The reported survival rates in previous studies were 68.8% for 1 year, 62.5% for 2 year, and 62.5% for 3 year (10); 82% for 1 year and 22% for 2 year (26); and 9% for 1 year and 5% for 2 year (36). In the latter study, the significant majority of the patients were managed non-surgically, highlighting the benefits of surgical management in both a palliative and OST context.

This study has several limitations that warrant consideration. The retrospective nature of the analysis limits control over data consistency and completeness, which may influence the reliability and comparability of the results. The small sample size will impact the power of the statistical analysis. Meaningful comparisons across existing studies are limited by varied protocols and heterogeneous data. However, future prospective studies could adopt standardised methods for assessing pre- and post-operative function, clinical outcomes, and histological classification. This would facilitate the integration of retrospective data from previous studies, enabling a larger pooled patient population and improved statistical power.

In conclusion, whilst Labrador Retrievers were over-represented in this study and may appear to be more susceptible to PNSTs than other breeds, this has not been statistically evaluated against the hospital patient population. A diagnosis of spinal PNST should be considered as a differential in middle-aged dogs presenting with single-limb lameness and muscle atrophy, as well as those with additional neurological signs. Histologically, most are low or intermediate grade tumours, although grade has a lower impact on survival time. There is increasing evidence that survival time is dependent on the ability to get clean margins, and this is largely dictated by the location of the lesion, with proximal lesions that require durotomy and rhizotomy having poorer outcomes. Compartmental resection has the benefit of maintaining or improving limb function; however, it is important to dissect enough grossly normal nerve to ensure distal margins, which is likely to lead to a considerably longer overall survival time. Even when surgical management is expected to be palliative rather than curative, the majority of patients can expect stable or improved comfort and function, a short duration of hospitalisation, a low rate or post-operative complications and a reasonable survival time.

## Data Availability

The original contributions presented in the study are included in the article/Supplementary material, further inquiries can be directed to the corresponding author.
