# Supplementary material for: Canine spinal peripheral nerve sheath tumours in 18 dogs (2014–2023): surgical management and long-term outcomes
Source: Front Vet Sci. 2025 Oct 1;12:1653812. doi: 10.3389/fvets.2025.1653812 (PMC12520905; doi:10.3389/fvets.2025.1653812)
Supplement: Supplementary file 1 [file Table_1.docx]

| Patient | breed | Age at presentation | Gender | weight in KG | Presenting signs | Arnoczky-Tarvin grade | Modified Griffiths grade | Duration of clinical signs | Imaging | Location | Lesion size  LxWxH in mm | Surgery performed | Duration of hospitalisation | Post-operative grade at post-op re-examination (AT / MG) | Histologic grade | Histologic margin | Disease free interval (days) | Overall survival time (days) | Death due to PNST | Other information |
| --- | --- | --- | --- | --- | --- | --- | --- | --- | --- | --- | --- | --- | --- | --- | --- | --- | --- | --- | --- | --- |
| 1 | Labrador Retriever | 6y11m | FN | 35 | Lameness, muscle atrophy | 3 | 1 | 2m | CTA, CT myelo | C8, T1  Extraforaminal | 100x35x15 | Compartmental resection | 1d | AT 2, MG 0 | 1 | R1 distal | 984 | 1357 | Yes | Pancarpal arthrodesis to improve carpal hyperflexion |
| 2a | Labrador Retriever | 8y11m | FN | 28.6 | Lameness, muscle atrophy | 2 | 0 | 3w | CTA, MRI | C8  Extraforaminal | 110x15x10 | Laminectomy, durectomy, rhizotomy | 3d | AT 2 | 1 | R1 distal | 258 | 326 | Yes | Second surgery patient 2b |
| 2b | Labrador retriever | 9y7m | FN | 33 | Lameness, paraesthesia, monoparesis | 2 | 2 | Staging for PCA | CTA  CT myelo | C7 intradural | 17x10x6 | Laminectomy, durectomy, rhizotomy | 6d | AT2, MG 2 | 1 | R1 proximal | 56 | 326 | Yes | Recurrence from R1 C8 margin extending into C7 noted at staging for PCA |
| 3 | Dandie Dinmont | 9y | FN | 10.8 | Ataxia and paraparesis | 0 | 2 | 2m | MRI, CTA | T6, intradural | 15x13x8 | Laminectomy, durectomy, rhizotomy | 2d | MG 0 | 2 | R1 proximal | 213 | 216 | Yes | Paresis recurred. Recurrence confirmed on CT myelo. |
| 4 | Labrador Retriever | 10y8m | MN | 30 | Lameness, Paraesthesia, muscle atrophy | 3 | 1 | 7m | CTA | T1 extraforaminal | 70x6x6 | Compartmental resection | 1d | AT 1, MG 0 | 1 | R1 distal | 155 | 566 | Yes | Lameness recurred at 155d. CT angio documented enlarged C8 nerve. |
| 5 | Rough Collie | 7y | ME | 24 | Lameness, muscle atrophy | 4 | 0 | 3m | CTA, MRI | L5 and femoral nerve, extraforaminal | 195x50x50 | Laminectomy, L5 neurectomy, coxofemoral amputation | 3d | n/a | 3 | R0 | 1374 | 1374 | No | Clavien-Dindo grade II UTI resolved with co-amoxyclav |
| 6 | Belgian Shepherd Dog | 12y3m | FN | 17.6 | Lameness, muscle atrophy, ipsilateral miosis | 4 | 0 | 1m | CTA | C8, extraforaminal | 60x10x6 | Laminectomy, durectomy, rhizotomy | 8d | AT 4, MG 2 | 3 | R1 proximal | 28 | 28 | Yes | Clients elected euthanasia due to lack of improvement and guarded prognosis |
| 7 | Border Terrier | 9y10m | ME | 9.8 | Lameness, paraesthesia | 3 | 1 | 3m | CTA, MRI | C8, T1, radial, extradural compression | 90x15x10 | Laminectomy, durectomy, rhizotomy, forequarter amputation | 2d | n/a | 2 | R1 proximal | 476 | 476 | Unknown, lost to further follow up. | DFI and OST are the based on the date of last client contact. Clavien-Dindo grade I transient urinary incontinence |
| 8 | Labrador Retriever | 6y8m | FN | 28 | Lameness, muscle atrophy | 3 | 0 | 2m | CTA | C7, C8, radial nerve | 90x40x15 | Laminectomy, durectomy, rhizotomy, forequarter amputation | 2d | n/a | 2 | R0 | 311 | 311 | Unknown | Euthanaised due to weight loss, no confirmed recurrence of clinical signs related to PNST |
| 9 | Labrador Retriever | 9y1m | MN | 26 | Lameness, muscle atrophy | 3 | 0 | 7w | CTA, MRI | C6, intradural | 60x25x15 | Laminectomy, durectomy, rhizotomy | Cardio-respiratory arrest at surgery | n/a | 3 | R1 proximal | 0 | 0 | Died at surgery | CLASSIC grade IV complication |
| 10 | Labrador Retriever | 7y9m | ME | 38 | Lameness, muscle atrophy | 2 | 0 | 6w | CTA, MRI | Sciatic | 55x30x40 | Hemipelvectomy | 1d | n/a | 2 | R0 | 1217+ | 1217+ | No, still alive at submission | Clavien-Dindo grade I transient urinary incontinence |
| 11 | Springer Spaniel | 10y4m | MN | 15.9 | Tetraparesis | 0 | 3 | 1w | MRI, CTA | C3, intradural | 60x40x30 | Laminectomy, durectomy, rhizotomy | 5d | MG 0 | 2 | R1 proximal | 157 | 157 | No | Died in his sleep at home with no recurrence of clinical signs |
| 12 | Labrador Retriever | 11y7m | ME | 26.7 | Lameness, muscle atrophy | 3 | 0 | 8w | CTA, MRI | Sciatic | 100x40x13 | Hemipelvectomy | 2d | n/a | 1 | R0 | 740 | 740 | No | Euthanaised for age related comorbidities |
| 13 | Bichon Frise | 7y9m | FN | 8.7 | Lameness, muscle atrophy | 2 | 0 | 5m | CTA | C8, T1, radial nerve, extraforaminal | 90x40x20 | Forequarter amputation | 1d | n/a | 1 | R0 | 961+ | 961+ | No, still alive at submission | EMG fibrillation potentials and positive sharp waves |
| 14 | West Highland White Terrier | 7y11m | FN | 10 | Lameness, muscle atrophy, monoparesis, incontinence | 3 | 2 | 4m | MRI, CTA | L7, S1, S2, sciatic nerve | 55x28x12 | Laminectomy, neurectomy, hemipelvectomy | 1d | n/a | 2 | R1 proximal | 217 | 217 | Yes, urinary and faecal incontinence failed to resolve post-op | Clavien-Dindo grade II UTI (e.coli and enterococcus) resolved with co-amoxyclav |
| 15 | Labrador Retriever | 7y6m | ME | 36 | Lameness, muscle atrophy, paraesthesia | 2 | 1 | 7w | CTA, MRI | C7, intradural | 65x23x12 | Laminectomy, durectomy, rhizotomy | 1d | AT  2, MG 0 | 2 | R1 proximal | 161 | 289 | Yes | Recurrence presumed but no imaging |
| 16 | Crossbreed | 7y6m | FN | 5.9 | Lameness, muscle atrophy | 3 | 0 | 1m | CTA | C8, T1, radial nerve, extraforaminal | 70x25x5 | Forequarter amputation | 1d | n/a | 1 | R0 | 443+ | 443+ | No, still alive at submission |  |
| 17 | Bedlington Terrier | 6y5m | FN | 12.7 | Lameness, muscle atrophy | 3 | 0 | 2m | CTA, MRI | C7 intradural | 52x7x7 | Laminectomy, durectomy rhizotomy | 1d | AT 0 | 2 | R1 proximal | 118 | 118 | Yes | Recurrence presumed but no imaging. |
| 18 | Cocker Spaniel | 8y2m | FN | 17.8 | Paraplegia | 0 | 4 | 2d | MRI, CTA | T12, intradural | 28x14x4 | Laminectomy, durectomy, rhizotomy | 3d | MG 0 | 2 | R1 proximal | 61 | 64 | Yes | Recurrence presumed but no imaging. |

Table 1. Patient data
